# A Second Sarah; Return of Menses in Old Age and Second Sight

**Published:** 1888-03

**Authors:** R. T. Knox

**Affiliations:** Vice President Texas State Medical Association; Gonzales, Texas


### A SECOND SARAH; RETURN OF MENSES IN OLD AGE, AND SECOND SIGHT.

LETTER FROM DR. R. T. KNOX, VICE PRESIDENT TEXAS STATE MEDICAL ASSOCIATION.

GONZALES, TEXAS, February 8, 1888.

*Editor Daniel's Texas Medical Journal:*

The case I am about to relate is one of just such singular import, I deem it would be injustice to the scientific world and to our beloved profession as well, to withhold; hence I relate the facts as have been obtained by me.

An old lady, aged 78 years, now and for several years past a resident of this place, a native of England, has all her life led, and is at this time leading a very active life in household affairs. General health very fine; while two years since she had a long spell of double pneumonia, which for time threatened to prove fatal, and happily did not, and left none of the ordinary sequels or bad results, she being restored to good health. At the age of thirty-five, after having borne only one child, the "menopause" came on with the usual marked disturbance, from which she rallied, but as then I thought was classed as an old woman, lost her sight, having to wear eye-glasses, etc. Some two years ago she abandoned the use of the spectacles and sees to read well with the naked eye. And now comes the strangest part yet. As of "Sarah of old" she became as other woman and at each succeeding month has her menses regularly, as she did when young, with no disturbance, and can do as much work as any one of her age and sex. It occurs to me, this is quite a peculiar case of both second sight and youth. I have heard of second sight before, and of cutting a set of teeth when very old, but nothing like as stated above.

Often during our epidemic of "dengue" several instances were called to my attention where parties several years before had ceased to have this trouble, claimed it had then returned on them, which, however, lasted but a short time. This case is subject to criticism, inquiries, etc., as I would like to hear from others, if like results were known to them. Yours,

R. T. KNOX.

